# Mcl-1 regulates reactive oxygen species via NOX4 during chemotherapy-induced senescence

**DOI:** 10.18632/oncotarget.15962

**Published:** 2017-03-07

**Authors:** Abeba Demelash, Lukas W. Pfannenstiel, Li Liu, Brian R. Gastman

**Affiliations:** ^1^ Department of Immunology, Lerner Research Institute, Cleveland Clinic, Cleveland, OH, USA; ^2^ Institutes of Head and Neck, Dermatology and Plastic Surgery, Taussig Cancer Center, Cleveland Clinic, Cleveland, OH, USA

**Keywords:** senescence, cancer therapy, Mcl-1, reactive oxygen species, NOX4

## Abstract

Mcl-1, a Bcl-2 family member, is highly expressed in a variety of human cancers and is believed to enhance tumorigenic potential and chemotherapy resistance through the inhibition of apoptosis and senescence. We previously reported that Mcl-1′s regulation of chemotherapy-induced senescence (CIS) is dependent on its ability to prevent reactive oxygen species (ROS) generation. In this report, we demonstrate that Mcl-1-regulated CIS requires not only ROS, but specifically mitochondrial ROS, and that these events are upstream of activation of the DNA damage response, another necessary step toward senescence. Mcl-1′s anti-senescence activity also involves the unique ability to inhibit ROS formation by preventing the upregulation of pro-oxidants. Specifically, we found that NADPH oxidases (NOXs) are regulated by Mcl-1 and that NOX4 expression in particular is a required step for CIS induction that is blocked by Mcl-1. Lastly, we illustrate that by preventing expression of NOX4, Mcl-1 limits its availability in the mitochondria, thereby lowering the production of mitochondrial ROS during CIS. Our studies not only define the essential role of Mcl-1 in chemoresistance, but also for the first time link a key pro-survival Bcl-2 family member with the NOX protein family, both of which have significant ramifications in cancer progression.

## INTRODUCTION

Bcl-2 family members are among the most important pro-oncogenic proteins in all forms of cancer [1, 2]. While the anti-apoptotic family members were thought to be somewhat interchangeable, relying heavily on a common binding cleft that sequesters and inhibits pro-apoptotic Bcl-2 family members and other pro-apoptotic proteins, studies investigating drugs targeting this binding cleft found important variations amongst these proteins [3, 4]. Mcl-1 in particular has proven difficult to target, and identifying an effective small molecule inhibitor of this cleft (or alternative methods of reducing Mcl-1 expression) is the subject of ongoing research [5–7]. Most studies suggest unique physical characteristics of the Mcl-1 anti-apoptotic binding cleft account for this difficulty in targeting [8].

We recently showed that Mcl-1 contains an additional unique domain that is distinct among Bcl-2 family members and critical for its known ability to inhibit chemotherapy-induced senescence (but not apoptosis) [9, 10]. This observation may explain why current targeted therapies do not completely inhibit Mcl-1 activity, as they do not account for this domain. We have further demonstrated that in cells lacking p53 (which are normally senescence-resistant), downregulation of Mcl-1 recapitulates senescence mechanisms downstream of p53, including upregulation of p21 and induction of reactive oxygen species (ROS). However, much of Mcl-1′s non-apoptotic, anti-senescence activities remain poorly understood.

In the current study, we demonstrate that targeting Mcl-1 during senescence-inducing doxorubicin treatment in otherwise senescence-resistant cells causes activation of the ROS-dependent DNA damage response (DDR), which is critical for the induction of senescence in cancer [11]. Consistent with our previous discoveries, we find that Mcl-1′s unique senescence-inhibiting domain is responsible for abrogating ROS production [10]. We further show that mitochondrial ROS is necessary for the induction of senescence. Although other Bcl-2 family members can regulate ROS, we found that Mcl-1 has a unique ability to prevent ROS not by upregulating anti-oxidants, as is the case for Bcl-2, but by preventing the upregulation of pro-oxidants [12]. We observe that Mcl-1 prevents the expression of NADPH oxidases (NOXs). Mcl-1′s inhibition of NOX4 specifically, limits its availability to translocate to the mitochondria and induce ROS. To our knowledge, this is the first documentation of any Bcl-2 family member regulating this class of proteins. As molecules like NOX4 and ROS themselves have critical functions in neoplasia, the ramifications of juxtaposing these pro-tumor factors will have great impact in better understanding how cancer evades current therapeutic regimens [13].

## RESULTS

### Mcl-1 inhibits DNA damage response (DDR) components to prevent CIS

**Figure 1:**
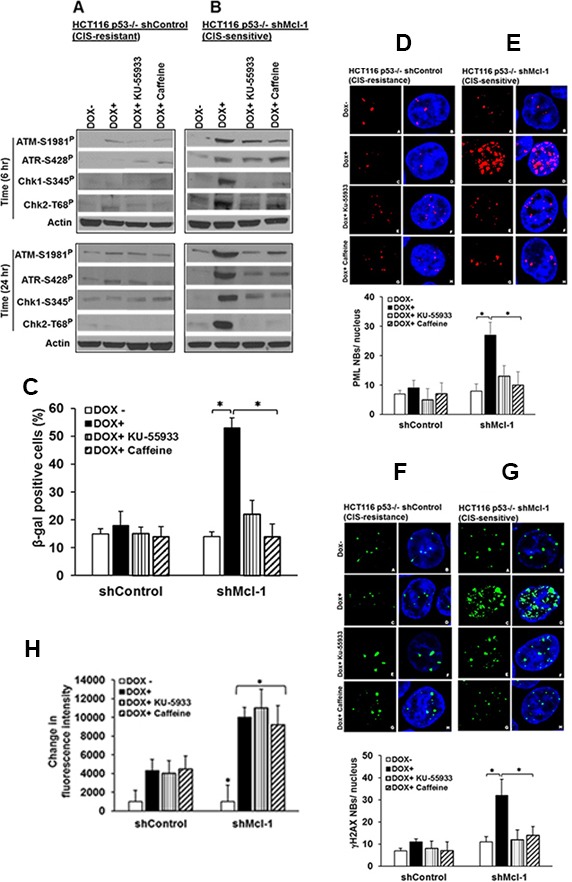
DDR components are important in Mcl-1-regulated senescence HCT116 p53−/− cells stably transfected with either a shControl (CIS resistant, Mcl-1 proficient) or shMcl-1 (CIS sensitive, Mcl-1 deficient), were treated with 100 ng/ml doxorubicin or left untreated in the presence or absence of caffeine (ATM/ATR inhibitor) or KU-55933 (ATM specific inhibitor). (**A** and **B**) Western blot detection of ATM-1981P, ATR-S428P Chk1-S345P, and Chk2-T68P under chemotherapy conditions. β-Actin (Actin) was used as a loading control. (**C**–**G**) Quantitative analysis of CIS in HCT116p53−/− shControl or shMcl-1 cells as assessed by β-gal activity (**C**), PML (**D** and **E**), and γ-H2AX nuclear body (**F** and **G**) formation in the presence or absence of the indicated ATM/ATR inhibitors. *P < 0.05, comparing those given DDR inhibitors + doxorubicin to doxorubicin alone. (**H**) The effects of DDR factor inhibitors on ROS generation. Cells were treated as in Figure A and B, and change in intracellular ROS production was determined using the Amplex Red reagent as described in the Material and Methods. Error bars represent ± S.D. Graphical data are inclusive of at least three independent experiments.

To better understand how Mcl-1 regulates chemotherapy-induced senescence, we started by examining a well-known component of this process, the activation of the DNA damage response (DDR), [14] Previous studies have demonstrated that Mcl-1 regulates and prevents DNA damage directly at the site of DNA breaks during apoptosis, as evidenced by co-immuno-precipitation of Mcl-1 with molecules such as γ-H2AX [15]. However, in our model of CIS, Mcl-1 prevents accumulation of these DNA damage factors, which would preclude direct binding as the only role for Mcl-1 [9, 10]. Figure 1A reveals that under CIS conditions induced by low-dose doxorubicin (DOX+) in CIS resistant, HCT116 p53^−/−^ (shcontrol) cells, there is little expression of activated DDR components (phospho-ATM, ATR CHK1/2). However, in Mcl-1 knock-down cells (shMcl-1, CIS-sensitive), doxorubicin treatment causes significant up regulation of all four DDR factors studied (Figure 1B). To test whether these DDR factors are important in the initiation of CIS due to the lack of Mcl-1, we treated the same paired cell lines (CIS sensitive and resistant) with 2 inhibitors of the DDR: caffeine, which inhibits both ATR and ATM; and the ATM-specific inhibitor KU-55933. Figure 1A and 1B reveals that both inhibitors were effective at preventing activation of all DDR components studied. Although ATM and ATR can regulate separate DDR pathways, our findings are consistent with recent reports that activated ATM can initiate ATR. [16] Using multiple assays for senescence we then found that in Mcl-1 knock-down cells (CIS-sensitive), both caffeine and KU-55933 were effective at preventing the induction of CIS (Figure 1C–1G).

We have previously demonstrated that ROS production is a key step in the induction of senescence [9]. As such, we next sought to determine whether DDR activation occurs before or after ROS production during CIS by observing whether DDR-inhibitor treatment could affect ROS production in Mcl-1 knock-down cells. Figure 1H demonstrates that although DDR inhibitor treatment prevents CIS, ROS production is not affected. These data indicate that Mcl-1′s inhibition of ROS production, critical for CIS abrogation, is upstream of DDR activation.

### A novel internal domain of Mcl-1 is required for its anti-ROS activity

**Figure 2:**
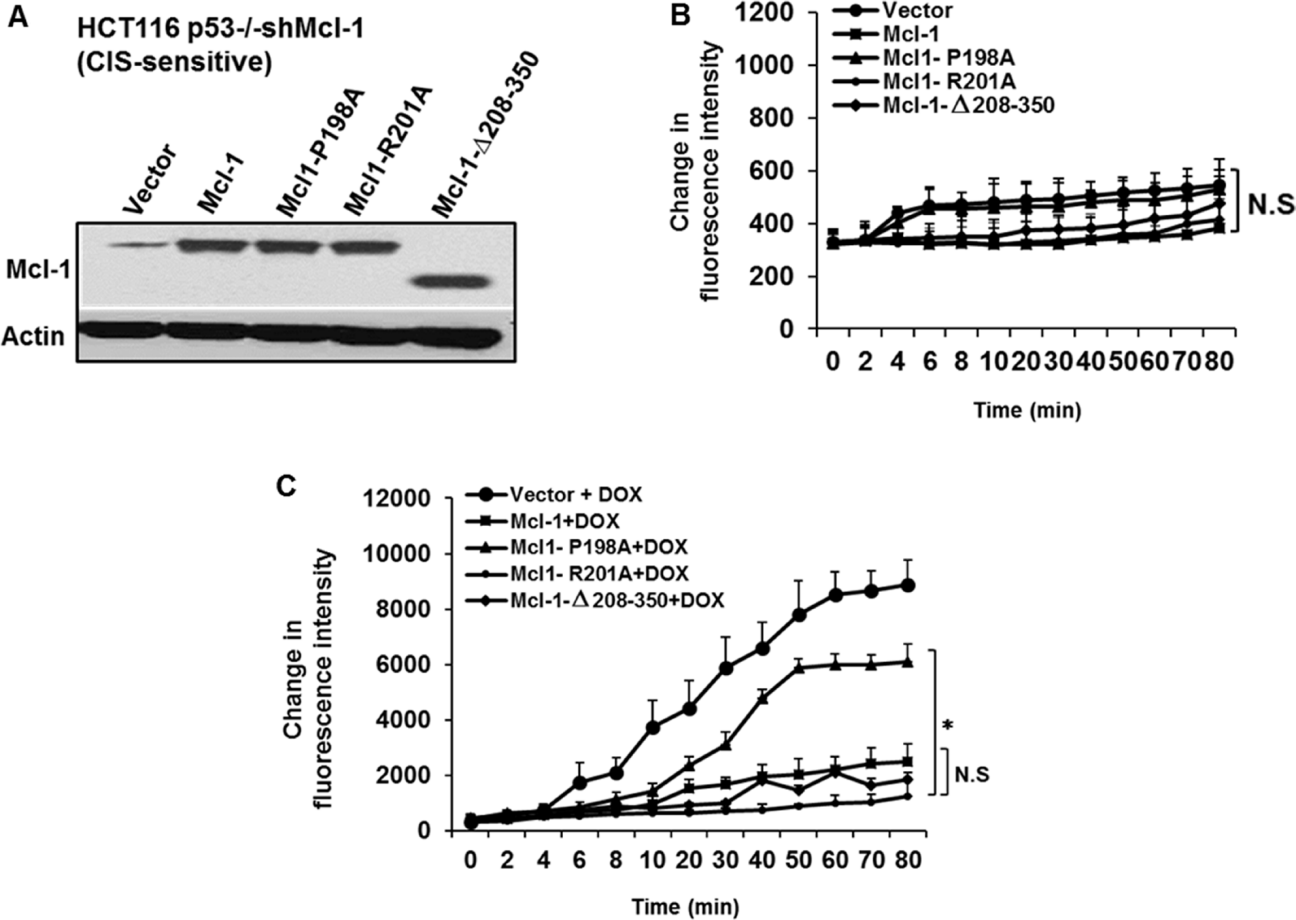
The Mcl-1 P198A residue required for inhibition of chemotherapy-induced ROS generation HCT116 p53−/− shMcl-1 cells were transiently transfected with vector control, wild type Mcl-1, or the following Mcl-1 mutants: P198A, R201A and Δ208–350 and were either left untreated or treated with doxorubicin. The ROS levels then were assessed with Amplex Red at the indicated time points. (**A**) Western blot of Mcl-1 protein levels after transfection of the indicated constructs. Cells expressing the indicated mutants were left untreated (**B**) or treated with doxorubicin (**C**). The data in (C) show that the expression of P198A residue but not the deletion of the BH3 region of Mcl-1 in HCT116 p53−/− shMcl-1 cells augmented chemotherapy-induced ROS production. The last time points (80 min) of ROS production measurements has been compered for the statistical differences between Mcl-1 mutants. Data are representative of three separate experimental cultures and transfections done in different days. Error bars represent ± S.D.

Anti- apoptotic Bcl-2 family members function in part by binding to pro-apoptotic BH3-only molecules through a canonical binding cleft [17]. Using extensive mutagenesis, we recently identified four specific residues within an undefined loop domain of Mcl-1 that are important for anti-CIS function both *in vitro* and *in vivo*. For example, an alanine substitution at residue 198 greatly reduced Mcl-1′s anti-CIS activities, while a similar substitution at residue 201 or a deletion of Mcl-1′s three BH domains (Δ208–350) did not [10]. Here we examined the effects of these mutants on Mcl-1 mediated anti-ROS activity after chemotherapy. These constructs were transiently expressed in HCT116 p53−/− cells with stable knock-down of Mcl-1 (CIS-sensitive). The expression of the constructs was confirmed by western blotting (Figure 2A). In untreated cells, expression of the mutants (or empty vector) did not cause a significant change in baseline ROS production (Figure 2B). After treatment with doxorubicin, however, increased ROS production was detected only in the mutant containing the alanine substitution at residue 198 (P198A) (Figure 2C). This is in contrast to constructs containing the R201A substitution or C-terminal BH3 domain deletion (Δ208–350), which had ROS levels similar to those in cells expressing wild type Mcl-1. These results show Mcl-1′s ability to regulate ROS production under CIS conditions depends on residues in the loop domain (and not its canonical C-terminal anti-apoptotic domain) also known to regulate its anti-CIS function [10].

### Mitochondrial ROS is significantly involved in induction of senescence

**Figure 3:**
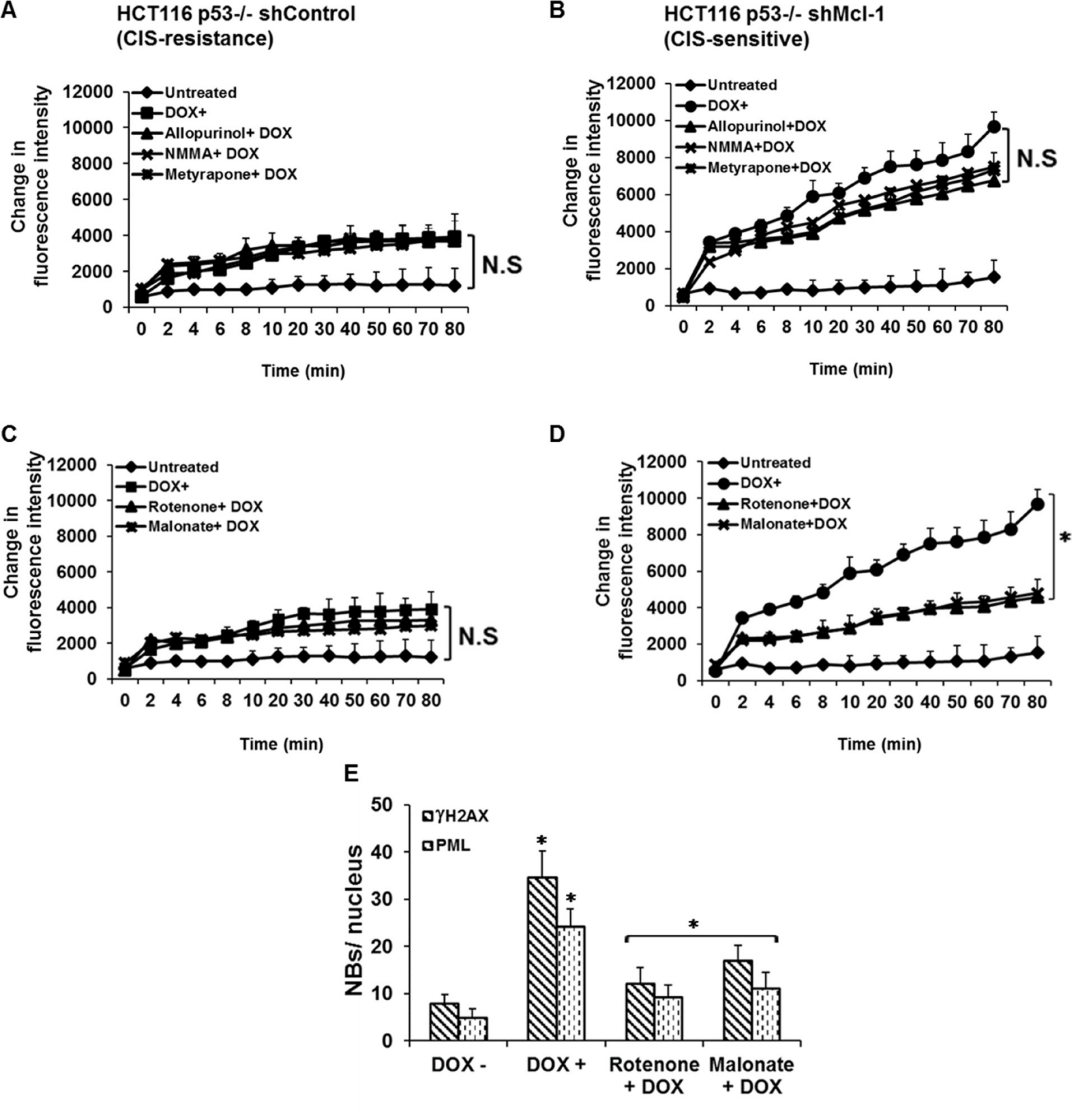
Mcl-1 acts to suppress mitochondrial ROS production thereby inhibiting CIS (**A**, **B**) Effects of cytoplasmic ROS-generating enzymes inhibitors on CIS. Cells were pretreated with or without the inhibitors: allopurinol (xanthine oxidase inhibitor); NG-monomethyl-L-arginine (NMMA) (a nitric oxide synthase) or metyrapone (a cytochrome P-450 inhibitor) before or after doxorubicin treatment. The data in A and (**C**) show that inhibition of mitochondrial and cytoplasmic ROS had no significant effect on the overall levels of ROS in CIS resistant cells after chemotherapy treatment. In CIS-sensitive cells, doxorubicin treatment results in dramatically higher levels of ROS in a time-dependent manner, which are not affected by cytoplasmic inhibitors (B). However, the Mitochondrial ROS inhibitors significantly reduced ROS production in CIS-sensitive cells (**D**). (**E**) Quantitative analysis of CIS in Mcl-1-deficient cells with or without the mitochondrial ROS-generating enzyme inhibitors as assessed by γH2AX and PML nuclear body formation. *P < 0.05, comparing those given the indicated inhibitors + doxorubicin to doxorubicin alone. Error bars represent ± S.D. Data are inclusive of at least three independent experiments.

Having determined that ROS production is upstream of DDR activation during CIS, we next determined the source of ROS generation within the cells. In cancer, ROS can result from activation of oncogenes, aberrant metabolism, and mitochondrial dysfunction [18–22]. We evaluated the effects of various pharmacological inhibitors on the rate of ROS production under CIS conditions. The inhibitors we chose target enzymes that produce both non-mitochondrial ROS: allopurinol (xanthine oxidase inhibitor), NG-monomethyl-L-arginine (NMMA, a nitric oxide synthase inhibitor), metyrapone (a cytochrome P-450 inhibitor); and mitochondrial ROS: rotenone (a complex I inhibitor) and malonate (succinate dehydrogenase inhibitor). Figure 3A and 3C show that inhibition of both mitochondrial and non-mitochondrial ROS had no effect on the overall levels of ROS in CIS resistant cells after chemotherapy treatment. In CIS-sensitive cells, doxorubicin treatment results in dramatically higher levels of ROS, which are not affected by non-mitochondrial inhibitors (Figure 3B). Mitochondrial ROS inhibitors, however, are able to significantly lower ROS production in CIS-sensitive cells (Figure 3D). Because rotenone and malonate affect mitochondrial electron chain transport complexes (ETC) I and II respectively, and because ETC complex III is also known as a major source of mitochondrial ROS, we conducted a similar study using antimycin A and found that it's use had little effect on mitochondrial ROS production.(Supplementary Figure 1). This observation is consistent with previously published studies demonstrating that the Q_i_ subunit of complex III affected by antimycin A inhibition is downstream of the Q_o_ subunit, which is the a site of superoxide generation that results in hydrogen peroxide generation within the mitochondria, and suggests that rotenone and malonate act by preventing electron flow to complex III where it can be used to generate ROS [23]. These results clearly demonstrate that mitochondria are involved in chemotherapy-induced ROS production in CIS sensitive cells. We further examined whether mitochondrial ROS inhibitors affect downstream markers of senescence. As shown in Figure 3E, there is a significant decrease in γ-H2AX and PML nuclear body formation in response to mitochondria complex inhibitors, demonstrating that ROS generated by mitochondria is a necessary component of CIS development.

### Mcl-1 has no effect on anti-oxidants for its anti-CIS activities

**Figure 4:**
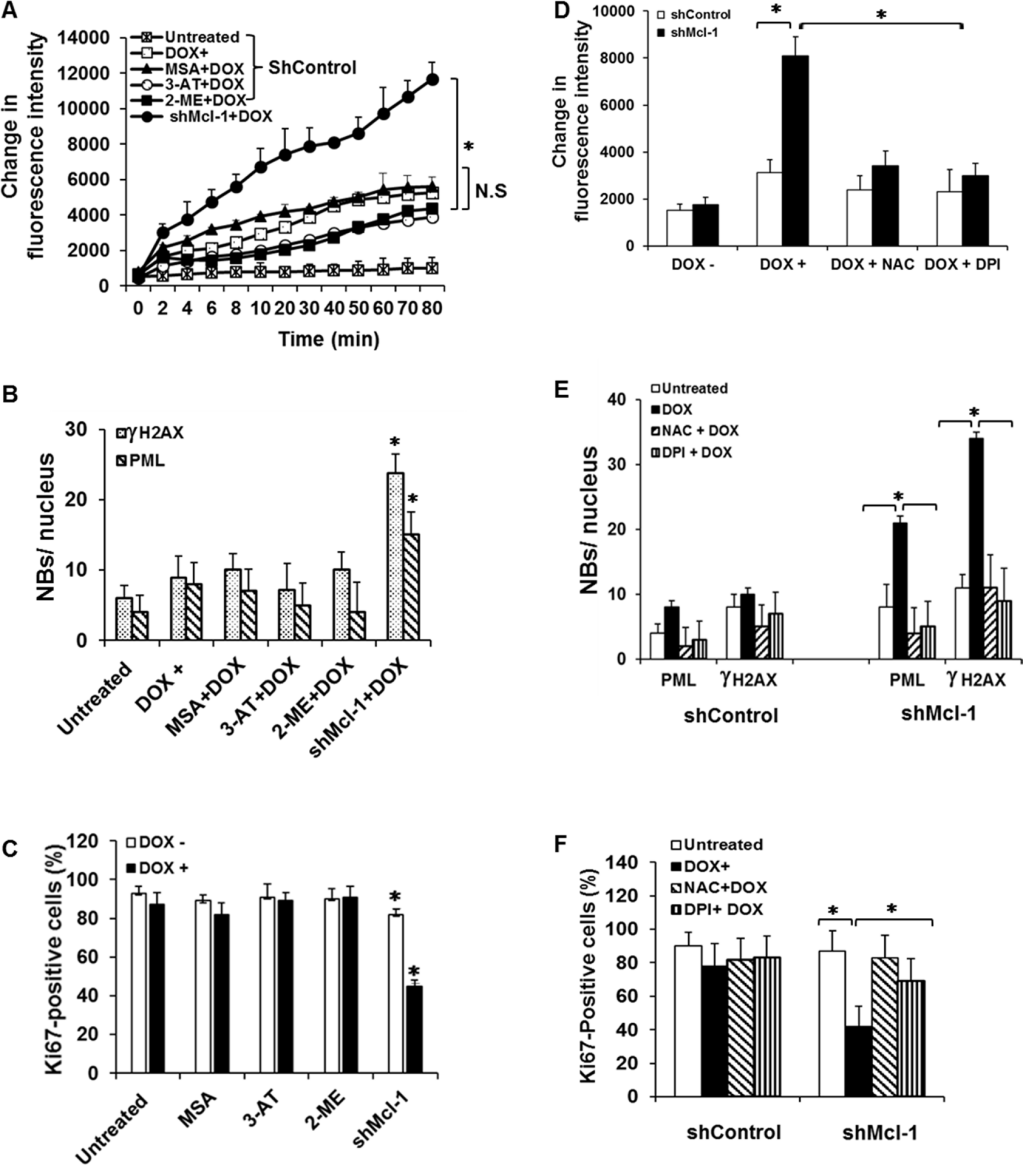
Mcl-1 does not inhibit CIS through up-regulation of anti-oxidant molecules (**A**) HCT116 p53−/− shControl or shMcl-1 cells were pretreated with inhibitor of catalase (3-AT); SOD (2-MT) or GPx (MSA) with or without doxorubicin. ROS levels were assessed with Amplex Red at the indicated time points. (**B** and **C**) Quantitative analysis of CIS in HCT116 p53−/− shMcl-1 cells as assessed by PML and γ-H2AX nuclear body formation (B) and Ki67 staining (C). (**D**–**F**) Reactive oxygen species inhibitors block CIS. HCT116 p53−/− shcontrol or shMcl-1 cells were pretreated with or without N-acetylcysteine (NAC) or diphenyleneiodonium (DPI) in the presence or absence of doxorubicin for 24 hours. (D) Effects of NAC and DPI on the ROS generation. (E and F) Quantitative analysis of CIS with or without ROS-generating inhibitors as assessed by PML and γ-H2AX nuclear body formation (E) and Ki67 staining (F). Significant differences are compared with untreated control versus doxorubicin treated as well as untreated versus doxorubicin plus inhibitors (*p ≤ 0.05). Error bars represent ± S.D.

Previous reports have demonstrated that other Bcl-2 family members are capable of inhibiting ROS generation through up-regulation of anti-oxidants or stabilization of the mitochondria [12, 24]. To assess if Mcl-1 regulates ROS generation through a similar up-regulation of anti-oxidants, we treated CIS-resistant/Mcl-1 proficient cells (HCT116 p53−/−) with chemical inhibitors of most major anti-oxidants, including: 3-AT (catalase), 2-MT (superoxide dismutase), and MSA (glutathione peroxidase) with or without doxorubicin. None of these inhibitors had a significant effect on Mcl-1′s anti-ROS function or senescence induction under CIS conditions as measured by PML and γ-H2AX foci formation or Ki67 staining (Figure 4A–4C). These results indicate that Mcl-1′s ability to prevent ROS-mediated CIS does not occur by up regulating anti-oxidants as is the case for Bcl-2. Knowing that the major processes leading to ROS generation are tightly regulated by a balance of anti- and pro-oxidants, we next tested the effect of diphenyleneiodonium (DPI), an inhibitor of the pro-oxidant family of NADPH oxidases (NOXs), as well as N-acetylcysteine (NAC), an anti-oxidant which we previously showed can prevent CIS [9]. Impressively, DPI (similar to NAC) caused a robust abrogation of ROS generation in Mcl-1 deficient cells as compared with the Mcl-1 proficient cells during CIS conditions after 24 hours of culture with doxorubicin, a time point that significant differences in ROS production can be observed (Figure 4D). DPI and NAC not only had a significant negative effect on ROS production, but also inhibited the induction of senescence (Figure 4E and 4F) in Mcl-1 deficient cells (CIS-sensitive) to the levels that were similar to CIS-resistant, Mcl-1 proficient cells. These data indicate that under CIS conditions, Mcl-1 inhibits the pro-oxidant side of ROS production, unlike other Bcl-2 family members.

### Mcl-1 prevents ROS generation and CIS through inhibition of NOX4 expression

**Figure 5:**
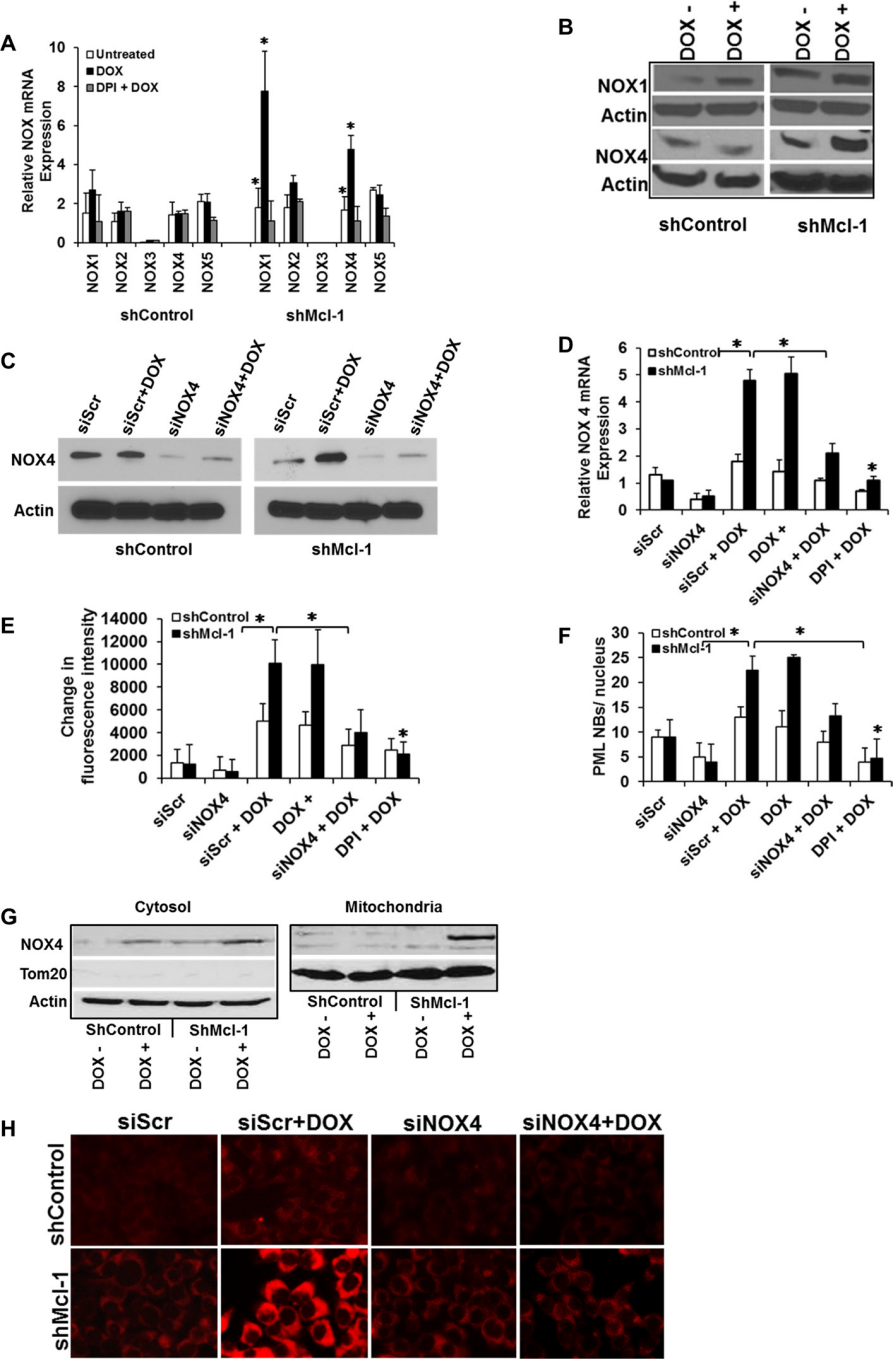
Mcl-1 prevents accumulation of NOX4, protecting cells from ROS-mediated CIS (**A**) Differential expression of the major NADPH oxidase (NOX) family members during CIS conditions. Cells were treated with doxorubicin or left untreated in the presence or absence of DPI. Levels of Nox1-5 mRNA were determined by real-time quantitative PCR (RT-qPCR). (**B**) NOX1 and NOX4 protein expression assed by western blot. (**C**–**F**) Mcl-1 prevents accumulation of NOX4 and protects cells from ROS-mediated CIS. Cells were transfected with scramble siRNA (siScr) as control or with NOX4 siRNA (siNOX4). After doxorubicin treatment, the level of NOX4 was quantified by western blotting (C). Cells transfected as in Figure C were treated with or without doxorubicin or doxorubicin + DPI after which the level of NOX4 mRNA was quantified by real-time qPCR analysis (D), ROS levels (E), and PML nuclear body formation (F). (**G**) Expression of NOX4 protein in mitochondrial and cytosolic fractions of Mcl-1 proficient (shControl) or deficient (shMcl-1) cells under CIS conditions. Fraction purity was verified by staining for Tom20 as a mitochondrial marker and loading control. β-actin was used as a loading control for the cytosolic fraction. Significant differences are compared with untreated control versus doxorubicin treated as well as untreated versus doxorubicin plus inhibitors (*p ≤ 0.05). Error bars represent ± S.D. Quantitative data are inclusive of at least three independent experiments. (**H**) Mitochondria ROS measurement by MitoSOX. Representative images of MitoSOX fluorescence. Microscopic imaging demonstrated markedly increased in mitochondrial fluorescence intensity of MitoSOX in NOX4 siScr cells treated with DOX for 24 h. The mitochondrial ROS generation where largely prevented by NOX4 siRNA (Figure 5H).

Although DPI is widely used to inhibit various NOXs, it can have broad inhibition of other flavoenzymes [25, 26]. Thus we next set out to both confirm and identify which NOX proteins induce ROS during CIS. We first assessed the mRNA expression of the five major NADPH oxidase family members by real-time RT-qPCR. Figure 5A shows major increases in NOX1 and NOX4 after doxorubicin treatment in Mcl-1 deficient cells only. Further, in the same cells, NOX4 protein expression was preferentially up-regulated during CIS as shown by western blot analysis (Figure 5B). These results prompted us to examine whether senescence resistance could be restored in Mcl-1 deficient cells through the knock-down of NOX4 expression. Two siRNAs (one shown) were designed to specifically silence NOX4 expression, and were used in shcontrol and shMcl-1 cells (as well as a scramble control). Successful knockdown of NOX4 with these specific siRNAs was confirmed at the protein (Figure 5C) and mRNA levels (Figure 5D). Under CIS conditions in sensitive cells (Mcl-1 deficient), knock-down of NOX4 was sufficient to significantly abrogate both ROS production (Figure 5E) and senescence induction (Figure 5F one of three senescence assays shown). Notably, knock-down of NOX1 by siRNA (Supplementary Figure 2A) did not affect senescence induction in sensitive cells (Supplementary Figure 2B, one of three senescence assays shown).

In Figure 3 we showed the critical nature of mitochondrial ROS in our model of CIS. As NOX4 was previously reported to localize to the mitochondria, we also examined its subcellular localization in our system of CIS [27–29]. Mitochondrial and cytosol fractions were prepared from CIS- sensitive and resistant cells under doxorubicin treatment and probed for NOX4. The purity of the fractionation procedure was confirmed by the presence or absence of Tom20 (a mitochondrial marker), which was also used as a protein loading control in the mitochondrial fraction, versus β-actin used in the cytosolic fraction (actin). Immunoblot analysis using NOX4 specific antibody shows that NOX4 is predominantly up regulated in the mitochondrial fraction in CIS-sensitive cells under doxorubicin treatment in the absence of Mcl-1 (Figure 5G, right panel). NOX4 levels were observed to a lesser extent in the cytosol. (Figure 5G, left panel). Thus in the absence of Mcl-1 in cells undergoing CIS, not only is NOX4 upregulated, but it is largely present in the mitochondria, explaining the critical ROS production observed. Interestingly, these studies also demonstrated that under doxorubicin treatment in the presence of Mcl-1 (ShControl cells), no detectable NOX4 is found in the mitochondria despite moderate expression in the cytosol, indicating a role for Mcl-1 in regulating the trafficking of NOX4 to the mitochondria, in addition to its effect on NOX4 expression. Finally, in order to demonstrate that the presence of NOX4 results specifically in increased mitochondrial ROS, we used MitoSOX, a fluorescent indicator of mitochondrial superoxide generation, to observe that knock-down of NOX4 expression in shMcl-1 cells results in decreased mitochondrial ROS generation after doxorubicin treatment (Figure 5H).

Thus we now show for the first time a direct link between a Bcl-2 family member and a specific NADPH oxidase and illustrate that Mcl-1 has a unique ability to inhibit ROS production by preventing the up regulation of the pro-oxidant NOX4, ultimately leading to CIS resistance. There may be other NOXs that Mcl-1 regulates, but these data are of great import given the key role both Mcl-1 and NOX4 play in carcinogenesis [30, 31].

## DISCUSSION

The targeting of survival proteins like Mcl-1 and the other Bcl-2 family members for cancer therapy is the subject of ongoing scientific and commercial interest [32]. While this interest has resulted in advances in treatment of hematogenous cancers, particularly in the inhibition of Bcl-2 and Bcl-xL in lymphoma, effective inhibitor treatments of solid tumors remains a challenge, particularly the effective targeting of Mcl-1 [33]. Indeed, the latest generation Bcl-2/Bcl-xL inhibitors are active at concentrations 10 fold lower than similarly designed Mcl-1 inhibitor, though a new generation of Mcl-1 inhibitor is under development that has higher activity [7, 33–38]. While initially defined as simply pro- or anti-apoptotic proteins, Bcl-2-family proteins are now known to interact with many other proteins in the cell, and contribute to the regulation of mitochondrial and endoplasmic reticulum function, autophagy, calcium homeostasis, and senescence [5, 39–44]. We have recently demonstrated that Mcl-1 is unique among the Bcl-2 family in that it can inhibit chemotherapy-induced senescence, even in the absence of p53, and that this inhibition occurs through a distinct molecular domain from its anti-apoptosis structure [9, 10].

In the current study, we expand upon our understanding of Mcl-1′s regulation of senescence by identifying its effects on well-known components of this process. We started by examining CIS induction of the DNA damage response (DDR). Although Mcl-1′s anti-apoptotic functions were previously linked to inhibition of the DDR by direct binding to DDR components, our data contrast with those results under CIS conditions in that we find Mcl-1 expression leads to lower expression of these components [15, 45]. We also demonstrate that chemicals designed to inhibit ATM and ATR can restore senescence resistance in cells made sensitive to CIS through knock-down of Mcl-1. By demonstrating that Mcl-1 inhibits the activation of major upstream DDR components (ATM, ATR, Chk1/2) and expression of downstream components like γ-H2AX, the very same proteins Mcl-1 was described to bind to, it is now clear that Mcl-1 may have a dual role: to directly bind and block activated components of the DDR (during apoptosis conditions), and to prevent them from ever being expressed (during senescence conditions) [15].

Our previous and current studies also highlighted that CIS pathways require generation of ROS and Mcl-1′s ability to inhibit CIS was dependent on its anti-ROS functions. ROS production in cancer is known to induce both cellular proliferation and injury [46–49]. Our study, however, showed that under senescence-inducing conditions, ROS production is upstream of other senescence-related events, including the DDR. Our observation that ROS production precedes the DDR during CIS is consistent with other studies showing that ATM can function as a redox sensor after DNA damage [50]. Thus Mcl-1′s inhibition of ROS prevents cellular injury and the DDR cascade.

We recently demonstrated that there is also a specific and unique domain within Mcl-1 that regulates CIS, distinct from those regulating apoptosis. Specifically there are 4 key residues in a loop domain (for instance P198) that, if mutated, will abrogate Mcl-1′s anti-senescent functions [10]. By re-expressing mutant Mcl-1 constructs in Mcl-1-deficient, CIS sensitive cells and measuring ROS production under CIS conditions, we were able to show that the P198 residue, (but not any of its C-terminal anti-apoptotic Bcl-2 homology domains) is necessary for anti-ROS function. Thus our data demonstrating that this internal loop domain is required to prevent ROS production are consistent with Mcl-1′s CIS-regulating activities.

Having identified that ROS is upstream of the DDR we delved further into the characteristics of its production. The generation of ROS is closely regulated by the balance of anti-oxidant and pro-oxidant actions, and members of the Bcl-2 family are known to affect generation of ROS under conditions of cellular stress [51, 52]. For instance, several Bcl-2 family members have been characterized as activators of anti-oxidants [50, 53, 54]. In contrast, we find that Mcl-1 affects ROS generation not by up regulation of anti-oxidants, but instead by regulating the expression of pro-oxidants, and specifically NOX4. To our knowledge, this is a unique finding among the Bcl-2 family. The NOX protein family are highly studied in cancer due to their role in inducing specific types and quantities of ROS, and the balance of their activities in relation to the pro-survival activities of the Bcl-2 family are not completely understood. Based on our data, Mcl-1 can be considered a regulator of this important pro-oncogenic class of molecules. Interestingly, we noted that doxorubicin treatment of shMcl-1 cells also results in the expression of NOX1, although subsequent knock-down of NOX1 resulted no effect on senescence. Both NOX1 and NOX4 expression are controlled by similar regulatory elements, so it is not surprising that expression of both genes are increased in our CIS model [55]. However, NOX1 appears to be a general cancer promoting factor, especially in CRC while NOX4 by itself was not shown to promote CRC progression [56, 57]. Additionally, of the two only NOX4 is known to induce liberation of H_2_O_2_ [28]. Thus, it is likely that NOX1 is upregulated to promote cancer progression during stress situations, while NOX4 is a factor that produces ROS which contributes to cellular injury and senescence [57]. How Mcl-1 specifically regulates the expression of NOX4 is under active investigation by our lab.

Our studies focused on production of H_2_O_2_ as the relevant species of ROS generated during the course of CIS. The reason for this is two-fold: 1) previous studies have indicated that while NOX4 generates significant levels of hydrogen peroxide, associated increases in superoxide radical generation are difficult to detect; and 2) CIS involves processes in various cellular compartments in addition to the mitochondria, including the DDR and gene expression changes in the nucleus, and H_2_O_2_ is a more stable molecule that is able to diffuse freely throughout the cell [58, 59].

While previous studies have found that inhibition of complexes I, II, and III of the mitochondrial electron transport chain all result in increased superoxide generation (especially complexes I and III), it is only inhibition of the Q_i_ site of complex III that results in increased hydrogen peroxide generation in intact mitochondria [23, 60–62]. These findings are similar to our own studies in doxorubicin-treated, shMcl-1 cells where use of roteneone and malonate decreased hydrogen peroxide ROS generation while antimycin A increased it; indicating that ROS generated from complex III likely represents the principle site of ROS generation during CIS. Whether the mitochondrial ROS represents an up-stream event that sets up a feed-forward process that leads to the traffic of NOX4 to the mitochondria further enhancing ROS production has yet to be determined.

Interestingly, we found that doxorubicin treatment alone was able to measurably increase cellular ROS, even in the presence of various ROS inhibitors or knock-down of NOX4. Doxorubicin is known to localize to the mitochondria, where it is directly reduced into superoxide radicals by interaction with complex I [63]. It may be that doxorubicin-treated cancer cells have an initial induction of ROS that triggers downstream processes of senescence and further ROS production, but in CIS-resistant cells these events (including further ROS production) are blocked by both the expression of Mcl-1 and absence of p53 - both common features of cancer. In normal cells, senescence-inducing therapies would not be hindered as there is no upregulation of Mcl-1 or loss of tumor suppressor genes. For example, one of the significant side-effects related to doxorubicin treatment is cardiac toxicity, which often limits its clinical effectiveness [64, 65]. In particular, NOX2 and NOX4 are both highly expressed in heart tissue, and contribute to ROS-related pathologies including tissue damage during congestive heart failure and cardiovascular disease, especially in aged tissue [66, 67]. The association of NOX4 with elevated ROS and age-related senescence in the heart is particularly interesting in that NOX4 overexpression induces cellular senescence in a similar manner to our model of doxorubicin-induced CIS, and doxorubicin can induce senescence in cardiac cells [68–70]. More relevant to Mcl-1 biology, the absence of Mcl-1 in cardiac tissue specific mouse genetic knock-out models is associated with abnormal mitochondrial function, inability to regulate autophagy, and cardiac failure [71, 72]. Although these studies did not evaluate the role of Mcl-1 and ROS in cardiac injury, other models of heart failure have demonstrated this association [73, 74].

It is well established that Bcl-2 family members which do not inhibit CIS do inhibit ROS generation through stabilization of the mitochondria [24]. In our study we found that the most important ROS during CIS, were those generated in the mitochondria. Importantly, the mitochondrion is the organelle most commonly associated with Mcl-1 apoptosis-related activities [5]. We now add to the list of Mcl-1′s unique stabilization of the mitochondria abilities by illustrating its unique capability to not only prevent NOX4 expression, but NOX4′s subcellular translocation to the organelle and subsequent ROS generation. This observation is consistent with other studies that have demonstrated NOX4 must be present in the mitochondria to induce oxidative stress and processes similar to senescence, especially in cancer cells [28, 29].

In summary, our work has expanded an understanding of Mcl-1′s ability to inhibit ROS and CIS. While ROS generation often contributes to transformation and oncogenesis, high levels are detrimental to cellular function, inducing apoptosis and senescence [75]. Specific targeting and enhancing of ROS production and/or inhibiting anti-ROS mechanisms are of increasing interest in designing the next generation of cancer therapeutics [22]. Cancer cells may have high baseline NOX family expression and ROS allowing for their genetic instability and cancer progression. Other cancer cells like we employ only have increased NOX expression and ROS production upon chemotherapy treatment. Considering that targeting NOX family members and/or ROS production is a bone fide and known strategy in cancer therapy development, understanding the gatekeepers of these events is critical to best tailor therapy for patient outcomes. Our study provides strong evidence that Mcl-1 is such a gatekeeper that could allow cells to keep NOX4 in check to prevent critical overloading of ROS and events like senescence. Moreover, as overcoming senescence is not just critical for cancer progression and treatment resistance, but impacts carcinogenesis in general, these results have major ramifications for cancer research and treatment strategies.

## MATERIALS AND METHODS

### Cell lines and culture conditions

HCT116 human colon cancer lines (p53−/−) were generously provided by Bert Vogelstein (Johns Hopkins University). The cell lines were maintained in Dulbecco's modified Eagle's medium (DMEM) supplemented with penicillin/streptomycin, non-essential amino acids, and 10% fetal bovine serum (FBS). HCT116 p53−/− shControl and HCT116 p53−/− shMcl-1 cells are derivatives of HCT116 p53−/− that stably express a transcript-specific short hairpin RNA (shRNA) that either knocks-down endogenous Mcl-1 expression or contains an irrelevant control RNA (Open Biosystems) and have been described previously [9]. All cell cultures were incubated at 37°C in a humidified incubator containing 5% CO2.

### Drug treatments

The drugs used were purchased from the following providers and used at the concentrations indicated: doxorubicin (100 ng/ml, Sigma); ATM specific inhibitor, KU-55933 (20 uM, Calbiochem); ATM and ATR inhibitor, caffeine (5 mM, MP Biomedical); anti-oxidant inhibitors: 3-amino-1,2,4-triazole (3-AT, a catalase inhibitor, 20 uM, Sigma-Aldrich); 2-methoxyestradio (2-MT, a SOD inhibitor, 10 uM, Sigma-Aldrich); mercaptosuccinic acide (MSA, a GPx inhibitor, 15 uM, Sigma-Aldrich); pro-oxidant inhibitor, a NADPH oxidase inhibitor diphenyleneiodonium (DPI, 30 uM, Sigma-Aldrich); antioxidant N-acetyl-L-cysteine (NAC, 5 mM, Sigma-Aldrich); cytoplasmic ROS-generating enzyme inhibitors: allopurinol (xanthine oxidase inhibitor, 100 uM, Sigma-Aldrich); NG-monomethyl-L-arginine (NMMA, a nitric oxide synthase, 100 uM, Sigma-Aldrich); metyrapone (a cytochrome P-450 inhibitor, 500 uM, Sigma-Aldrich); the mitochondrial ROS-generating enzyme inhibitors: rotenone (a complex I inhibitor, 10 uM, Sigma-Aldrich), malonate (succinate dehydrogenase inhibitor, 5 uM, Sigma-Aldrich), or antimycin A ( a complex III inhibitor, 20 uM, Sigma Aldrich).

### Plasmid transfections

Transient plasmid transfection in HCT116 p53−/− shMcl-1 cells was performed using Lipofectamine 2000 (Life Technologies) according to the manufacturer's instructions. Briefly, 2 × 10^5^ cells/well in 6 well plates or 1 × 10^5^ cells/well in 6 well plates on poly-L- lysine coated glass coverslips were transiently transfected with 0.5 ug of wild-type Mcl-1, P198A, R201A, Δ208–350 expressing constructs, or empty pcDNA3.1 vector (Invitrogen). Medium was changed after 24 hours and then cells were incubated for 48 hours prior to verifying transgene expression by western blotting. 48 hours post-transfection, cells were left untreated or treated in fresh media containing doxorubicin to induce senescence. For knock-down of NADPH oxidase 4 (NOX4) and (NOX1) by siRNA, HCT116 p53−/−shControl or shMcl-1 cells were transfected with NOX4 and NOX1 specific siRNA or with a negative control scramble siRNA (siScr) (Santa Cruz Biotechnology) and were cultured for 48 hours. After 24 hours doxorubicin (100 ng/ml) treatment, the gene and protein expression of NOX4 and NOX1 were quantified by real time quantitative PCR (RT-qPCR) and western blotting.

### Immunoblotting

Western blotting analyses were performed as described previously (Demelash et al. 2015). The membranes were visualized using enhanced chemiluminescence (ECL) reagents (GE Healthcare) or WesternBright Quantum kit (Advansta, Menlo Park, California, USA). The following antibodies (at the indicated dilutions) were used in this study: anti-NOX1 (rabbit, 1:500, Santa Cruz Biotechnology); anti-NOX4 (rabbit, 1:1000, Santa Cruz Biotechnology); anti-Mcl-1 (rabbit, 1:1000); anti-phospho-ATM Ser198 (ATM-S1981^p^, rabbit, 1:500); anti-phospho-ATR Ser428^p^ (ATR-S rabbit, 1:500); anti-phospho-Chk1Ser345^p^ (rabbit, 1:500); anti-phospho-Chk2T68^p^ (rabbit 1:500). Antibodies were purchased from Cell Signaling Technology. Mouse anti-β-actin (Santa Cruz Biotechnology) at a dilution of 1:10000 was used as loading control.

### Immunofluorescence

Immunofluorescence was performed as described previously [10]. Anti-PML (mouse, 1:100 dilution) was purchased from Santa Cruz Biotechnology. Anti-γH2AX (Ser-139, mouse, 1:100) was purchased from BioLegend, and anti-Ki67 (mouse, 1:100) was from BD Biosciences. Cells were incubated with a goat anti-mouse (clone Poly 4043) or a donkey anti-rabbit (clone Poly 4064) secondary antibody conjugated with Cy3 (BioLegend, San Diego, CA) for 1 h in the dark, washed with PBS, and mounted on microscope slides using Vectashield mounting medium containing DAPI for fluorescence (Vector Laboratories, Burlingame, CA). Images were captured on a Leica SP2 confocal microscope using the appropriate filter sets.

### ROS measurement

Changes in intracellular ROS production were determined using the Amplex Red reagent (10-acetyl-3, 7 dihydroxyphenoxazine) using the protocols provided by the manufacturer (Molecular Probes, Eugene, OR). Approximately 10.000 HCT116 p53−/−shControl or shMcl-1 cells/well were allowed to grow in 96 well plates for 24 hours. The following day, the media were replaced and the cells were pre-incubated for 1 hour with ATM specific inhibitor, KU-55933; ATM and ATR inhibitor, caffeine; anti-oxidant inhibitors: mercaptosuccinic acid (MSA, a GPx inhibitor); 3-amino-1,2,4-triazole (3-AT, a catalase inhibitor) or 2-methoxyestradio (2-MT, a SOD inhibitor); a pro-oxidant inhibitor, a NADPH oxidase inhibitor (DPI); the antioxidant N-acetyl-L-cysteine (NAC); cytoplasmic ROS-generating enzyme inhibitors: allopurinol (xanthine oxidase inhibitor); NG-monomethyl-L-arginine (NMMA, a nitric oxide synthase), metyrapone (a cytochrome P-450 inhibitor); the mitochondrial ROS-generating enzyme inhibitors: rotenone (a complex I inhibitor) or malonate (Succinate dehydrogenase inhibitor). The cells were then incubated in the presence or absence of doxorubicin. Fifty microliters of the Amplex Red reaction mixture (100 μM Amplex Red, and 0.2 U/ml horseradish peroxidase) was added to each well followed by 30 min incubation at 37°C. Amplex Red conversion to resorufin was measured at 590 nm emission and 560 nm excitation using a microplate reader. To measure specific mitochondrial generated ROS, MitoSOX Red was used as described in the manufacturer's protocol (Invitrogen). The fluorescent probe MitoSOX selectively reacts with superoxide in the mitochondria. Phenol red-free medium was used to avoid dye interference.

### Real-time qPCR

Total RNA was extracted from HCT116 p53−/− shControl or shMcl-1 cells using the RNeasy Mini Kit (Qiagen). cDNA was synthesized from 1 μg of RNA using the Super Script III First-strand Synthesis System Kit (Invitrogen) according to the manufacturer's instructions. mRNA levels were determined by Real-time quantitative PCR (RT-qPCR) using SYBR Green and Taq Master Mix kit (Qiagen) with a set of primers specific for the human NOX family (NOX1 to NOX5). The threshold cycle (Ct) value of the target gene was normalized to the expression of reference gene GAPDH to obtain a 2 −ΔΔCt value. All reactions were performed in triplicate.

### β-galactosidase senescence assays

Following 6 days of treatment or no treatment with drugs, cells were assayed for senescence-associated beta-galactosidase (SA-β-gal) expression as previously described [76]. Briefly, cells were washed and fixed with 2% PFA (Fisher Scientific) for 5 minutes at room temperature. Cells were then incubated in the dark for up to 16 hours in a staining solution containing 1 mg/ml X-Gal (Gold Biotechnology) in dimethylformamide (Acros Organics), 40 mM of a 0.2 M citric acid/Na phosphate buffer pH 6.0, 5 mM potassium ferrocyanide (Sigma), 5 mM potassium ferricyanide (Sigma), 150 mM sodium chloride, and 2 mM magnesium chloride. Stained cells were then visualized under an inverted bright-field microscope. Ten representative fields were randomly selected for the quantification of β-gal positive cells as a percentage of the total cell number.

### PML and γH2AX foci quantification

Ten representative fields were randomly selected for the quantification of PML and γH2AX nuclear body formation (NBs). The numbers of foci present in each cell nucleus were manually counted in 30 transfected and drug treated cells as well as in transfected but not drug treated cells using a Leica DM5500 B florescent microscope at 40× oil immersion.

### Cell proliferation assays

The proliferative capacity of cells was determined by Ki67 immunohistochemical staining. Ten representative fields were randomly selected for the quantification of Ki67-positive cells. The numbers of Ki67-positive cells were manually counted in drug treated or untreated cells using a Leica DM5500 B florescent microscope at 40× oil immersion.

### Mitochondria and cytosol fractionation

Mitochondria and cytosol fractions were obtained using the Mitochondria Isolation Kit (Product No. 89874) for cultured cells from Thermo Scientific. Briefly, after 48 hours with or without 100 ng/ml doxorubicin treatment, 2 × 10^7^ HCT116 p53−/− shControl and HCT116 p53−/− shMcl-1 cells were harvested by centrifuging at ~850 × g for 2 minutes, and mitochondria were isolated following the protocol provided by the kit. Mitochondria pellets and whole cell lysates were lysed in RIPA buffer. After removing the insoluble material by 14,000 × *g* centrifugation, protein from mitochondria, and cytosol was quantified by Thermo Scientific BCA Assay kit (Product No. 23225). For gel electrophoresis, 30 ug of total protein was loaded per lane and separated by SDS-PAGE, and then transferred to PVDF membrane (Bio-Rad). For immunoblot experiments, the membranes were sequentially blotted with anti-NOX4 (Santa Cruz), anti-Tom20 (Santa Cruz), mouse β-actin (Santa Cruz) primary antibodies, and horseradish peroxidase-conjugated secondary antibody (Bio-Rad), followed by chemiluminescence visualization (PerkinElmer Life Sciences).

### Statistical analysis

The significance of difference between two groups were determined using Student's *t* test. Statistical evaluation of the data with multiple treatments was performed by one-way ANOVA for multiple comparisons. In all cases, *P* values of < 0.05 were considered significant. Data are presented as mean ± standard deviation (SD) from at least three independently and separately conducted experiments.

## SUPPLEMENTARY MATERIALS FIGURES AND TABLES



## Abbreviations

CIS: Chemotherapy-induced senescence
DDR: DNA damage response
DPI: diphenyleneiodonium
NAC: N-acetylcysteine
NMMA: NG-monomethyl-L-arginine
ROS: Reactive oxygen species
