# Supplementary material for: Mcl-1 regulates reactive oxygen species via NOX4 during chemotherapy-induced senescence
Source: Oncotarget. 2017 Mar 7;8(17):28154–68. doi: 10.18632/oncotarget.15962 (PMC5438639; doi:10.18632/oncotarget.15962)
Supplement: Supplementary file 1 [file oncotarget-08-28154-s001.pdf]

Mcl-1 regulates reactive oxygen species via NOX4 during chemotherapy-induced senescence

Supplementary Materials

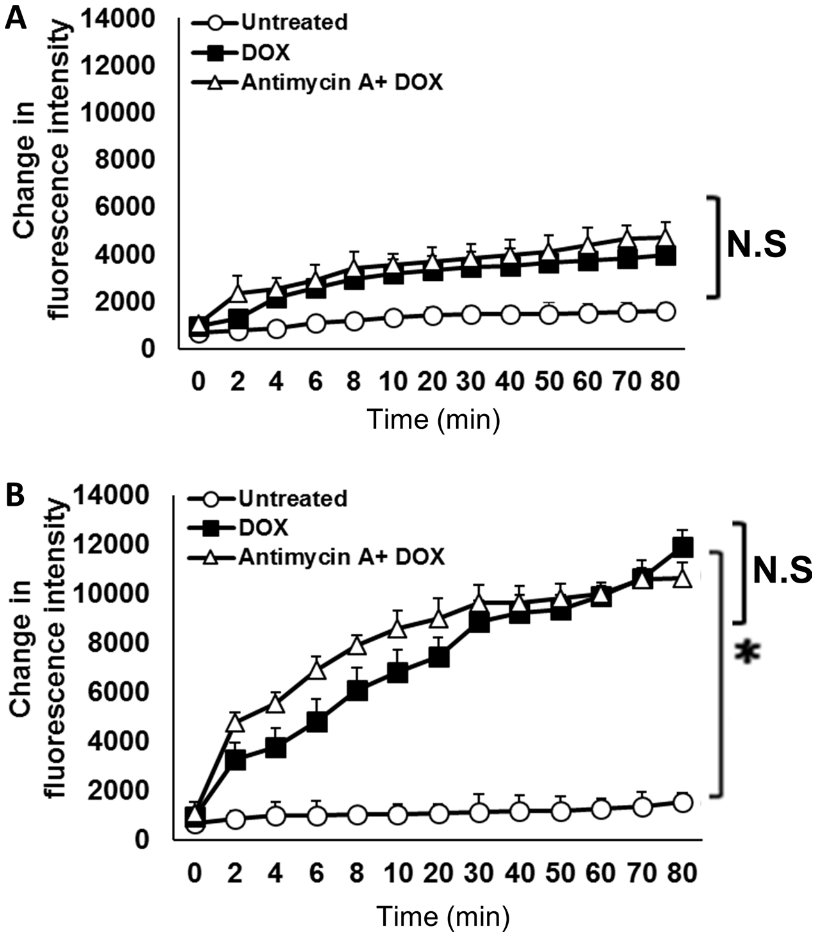

Supplementary Figure 1: Antimycin A , a complex III blocker increased the ROS generation in response to chemotherapy. HCT116 p53−/− shcontrol (**A**) or shMcl-1 (**B**) cells where treated in the presence or absence of Antimycin A and/or doxorubicin. After treatment, the ROS levels were assessed with Amplex Red at the indicated time points. N.S. indicates no statistical difference between doxorubicin-alone and doxorubicin plus antimycin A treatment groups at the final timepoint.

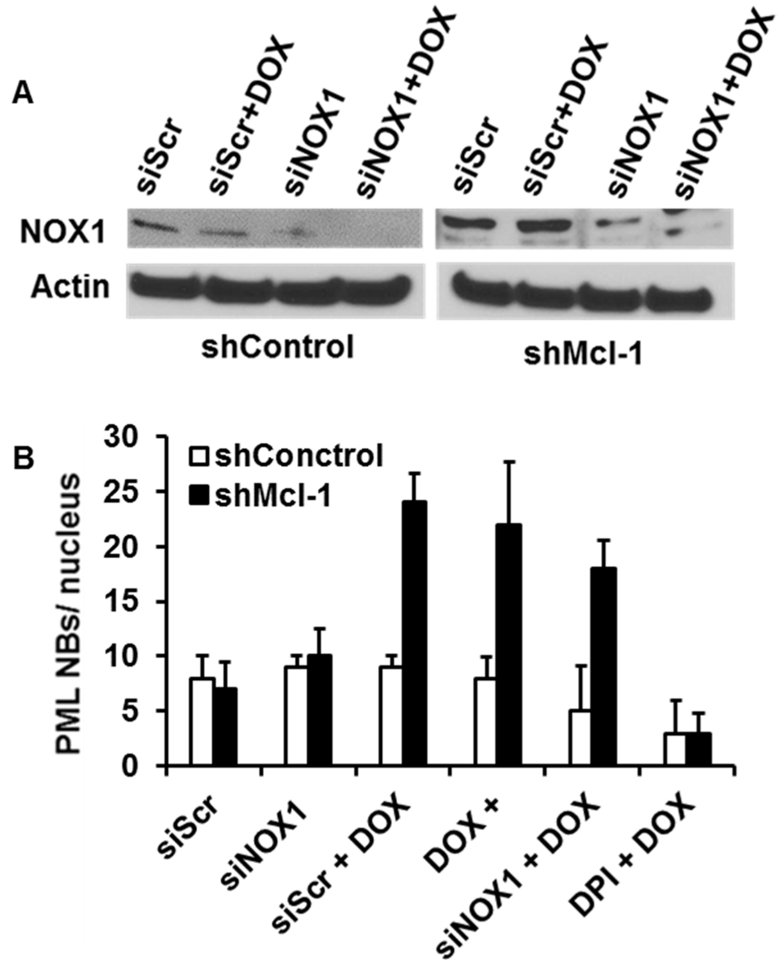

Supplementary Figure 2: Knock-down of NOX1 did not affect senescence induction in sensitive (Mcl-1 deficient) cells. HCT116 p53−/− shControl or shMcl-1 cells were transfected with scramble siRNA (siScr) as control or with NOX1 siRNA (siNOX1). After doxorubicin treatment, the level of NOX1 was quantified by western blotting (**A**). Cells transfected as in (A) were treated with or without doxorubicin or doxorubicin + DPI after which the level of PML nuclear body formation was quantified (**B**). Significant differences are compared with untreated control versus doxorubicin treated as well as untreated versus doxorubicin plus inhibitors. Quantitative data are inclusive of at least three independent experiments.
